# High-Cutoff Hemodialysis Therapy for Patients with Light Chain Cast Nephropathy and AKI Requiring Dialysis: PRO

**DOI:** 10.34067/KID.0000000000000151

**Published:** 2023-05-22

**Authors:** Sheron Latcha, Nelson Leung

**Affiliations:** 1 Department of Medicine, Renal Service, Memorial Sloan Kettering Cancer Center, New York, New York; 2 Department of Hematology and Nephrology and Hypertension, Mayo Clinic, Rochester, Minnesota

**Keywords:** dialysis, acute kidney injury, hemodialysis, high cuff off hemodialysis, light chain cast nephrophathy, myeloma cast nephropathy

Advances in myeloma-directed therapy in the recent decades have improved the median survival from 3 to 8–10 years.^1^ Although newer agents have convincingly improved multiple myeloma outcomes, all drugs take days to weeks to begin lowering serum free light chain (sFLC) levels. Clinical data indicate that high cutoff hemodialysis (HCO HD) rapidly reduces sFLC levels, and this rapid reduction is associated with better renal outcomes.^2,3^ Patients with cast nephropathy are more likely to recover renal function if there is a 50% decrease in sFLC concentrations.^4^ Intuitively then, HCO HD should acutely lower sFLC levels in the interval it takes for the antimyeloma drugs to have a therapeutic effect and would thus be expected to improve dialysis independence. The results of the Studies in Patients With Multiple Myeloma and Renal Failure Due to Myeloma Cast Nephropathy (MYRE) and European Trial of Free Light Chain Removal by Extended Haemodialysis in Cast Nephropathy (EuLITE) trials were a surprise and disappointment to clinicians who had eagerly anticipated confirmation that HCO HD would allow patients with multiple myeloma and AKI requiring HD to achieve dialysis independence. These two randomized, prospective, controlled clinical trials compared HCO HD with standard HD among patients with newly diagnosed biopsy-proven myeloma cast nephropathy. Earlier case series and retrospective studies which had shown that up to 80% of patients treated with HCO HD achieved dialysis independence compared with historical data showing that 30% of those treated with standard HD achieved this end point.^2^ By dissecting the methodology of MYRE and EuLite (Table 1), we aim to demonstrate that these studies did not definitively rule out a role for HCO HD in those who present with dialysis requiring AKI. Observed outcomes in these trials were limited by insufficient patient recruitment, imprecise randomization and inattention, and timely intervention for HCO HD-related complications.

**Table 1.** Limitations of MYRE and EuLite

Factor	MYRE	Potential Limitation
Primary end point study design	Dialysis independence at 3 mo. Study powered to detect a dialysis independence rate of 60% in HCO HD	Study may have been underpowered because newer therapies had already achieved dialysis independence in close to 50% patients
Insufficient time to achieve the primary end point	Prespecified interval was 3 mo	Dialysis independence increased over time in the HCO HD group: 3 mo 41%; 6 mo 56.5%; 12 mo 61%
Groups not well-randomized at baseline	Patients in the HCO HD group had more comorbidities at baseline	Precipitating factors for AKI at baseline in HCO HD versus HD groups were: Infection 11% versus 4% Hypercalcemia 20% versus 8% Dehydration 13% versus 10% NSAID use 39% versus 31%
Prerenal insults from HCO HD treatment	Albumin loss from the HCO HD membrane	Albumin supplementation required in 41% in HCO HD and 4% in HD

Factor	EuLite	Potential Limitation
Baseline AKI	Reversible causes not identified and treated before randomization	Intensity and frequency of HCO HD may have worsened any preexisting prerenal causes of AKI at baseline
Immunoglobulin loss with HCO HD	Immunoglobulin loss with HCO HD may explain the increased infection rate in the HCO HD group (58% versus 39%)	Higher rate of grade 3–5 infections in the HCO HD group may have led to interruptions for chemotherapy treatment

HCO HD, high cutoff hemodialysis; NSAID, non-steroidal anti-inflammatory drug.

The primary end point in MYRE was dialysis independence at 3 months. The study was powered to detect a dialysis independence rate of 60% in the HCO HD group compared with the HD group. This was based on previous data that showed that hemodialysis independence rates as high as 60% had been achieved with HCO HD.^2,5–7^ A closer inspection of these preceding reports on which the MYRE end points were based indicate that renal outcomes had already significantly improved with newer myeloma-directed therapy alone, and so MYRE may not have been appropriately powered to detect the additional effects from HCO HD. Before the introduction of proteosome inhibitors in 2005 and thalidomide and lenalidomide in 2006, fewer than 25% of patients with biopsy-proven cast nephropathy and dialysis-dependent renal failure achieved dialysis independence.^2^ Bortezomib-based triplet therapy without HCO HD has been reported to achieve dialysis independence in 48% of patients.^8^ In EuLite, all patients received triplet chemotherapy and 51% of the HD group achieved dialysis independence versus 25% as expected in the statistical plan. In the study of HCO HD versus retrospective HD control patients by Gerth *et al.*,^5^ 76.2% of patients in the HCO HD group were treated with bortezomib, thalidomide, or lenalidomide compared with only 25.5% of patients in the conventional HD arm. The observation in this cohort that 64.3% of patients in the HCO HD group achieved dialysis independence compared with 26.4% in the HD group may have been in part due to the use of newer and more effective chemotherapy.

In MYRE, hematologic response at 3 months was significantly different between the HCO HD and HD groups (89.1% versus 62.5%, *P* 0.003). Previous studies have shown that those who achieve earlier hematologic response have better renal outcomes. Thus, the HCO HD group would have been expected to have better renal outcomes on the basis of better hematologic response at 3 months. In addition to being underpowered for the primary end point, MYRE may not have allowed sufficient time to achieve the primary end point at the prespecified interval of 3 months. Indeed, dialysis independence in the HCO HD group improved over time: at 3 months, 41% of HCO HD versus 33% of HD (*P* = 0.42); at 6 months, 56.5% in the HCO HD versus 35% of HD (*P* = 0.04); and at 12 months, 61% in HCO HD versus 37.5% of HD (*P* = 0.02). Serial improvement in eGFR from the time of first HCO HD to 3, 6, and 12 months was similarly observed by Curti *et al.*^7^

One reason that those in the HCO HD group may have required a longer period to recover renal function is that those randomized to the HCO HD in MYRE seemed to have more factors contributing to kidney injury at baseline compared with those in the HD group. Precipitating factors for renal injury at baseline in the HCO HD group versus HD were infection (11% versus 4%), hypercalcemia (20% versus 8%), dehydration (13% versus 10%), and non-steroidal anti-inflammatory drug use (39% versus 31%). In addition, the HCO HD procedure itself may have contributed to ongoing prerenal insults after randomization, which would delay renal recovery. HCO HD membranes allow the removal of proteins up to 65 kDa and are associated with significant albumin loss,^9^ which is, in turn, clearly associated with hemodynamic consequences. In MYRE, albumin supplementation was required in 41% of HCO HD treatments versus 4% of HD sessions. With the caveat of a type 1 error, the observation that the HCO HD group had a trend to greater dialysis independence at 6 and 12 months could be a signal that HCO HD-treated patients had better renal outcomes despite more severe kidney injury at baseline and repetitive treatment-associated prerenal insults.

Similarly, the study design of EuLite likely affected study outcomes. Unlike in MYRE, patients in EuLite were not treated for reversible causes of AKI or given corticosteroids before randomization. Thus, patients who may not have required HD at all could have been inappropriately randomized for HCO HD and to the risks of prerenal and infectious complications inherent to intensive extracorporeal therapy. In EuLIte, mortality at 24 months was 37% in the HCO HD group versus 19% in the HD group. While the HD group received HD as determined by the treating nephrologist, the HCO HD group by protocol received daily 6–8 hours sessions for 9 of 11 days using two 1.1-m^2^ HCO dialyzers in series and then on alternate HCO HD days for 10 days. Possibly due to immunoglobulin losses, the rate of grade 3–5 infections was higher in the HCO HD versus HD group (58% versus 39%). Because infections usually lead to interruptions in chemotherapy, the HCO HD group likely had more frequent interruptions in treatment. Prior observations with HCO HD have shown that patients who received uninterrupted chemotherapy were able to achieve sustained reductions in sFLC.^2^

HCO HD filters are not yet approved for use in the United States. An opportunity exists to conduct an adequately powered multicenter clinical trial using modern myeloma-directed chemotherapy regimens to evaluate whether there is any benefit of HCO HD to achieve dialysis independence. Lessons learned from MYRE and EuLite include identifying and treating those with reversible causes of AKI before randomization, the preemptive use of albumin to avoid intra-HCO HD hypotension, prophylactic use of antibiotics to decrease infection rates, and individualized prescriptions in the HCO HD group on the basis of serial sFLC levels. HCO HD with chemotherapy is cost-neutral^7^ to the health care system but of potentially great benefit for patient survival and quality of life if it is proven to increase dialysis independence.
